# Supplementary material for: APOE ε4-Allele in Middle-Aged and Older Autistic Adults: Associations with Verbal Learning and Memory
Source: Int J Mol Sci. 2023 Nov 5;24(21):15988. doi: 10.3390/ijms242115988 (PMC10650864; doi:10.3390/ijms242115988)
Supplement: Supplementary file 1 [file ijms-24-15988-s001.zip › ijms-2670718-supplementary.pdf]

Supplementary Table S1. Additional Participant Demographics.

	ASD (n; % of sample)		NT (n; % of sample)	
	ε4-allele Non-carriers	ε4-allele Carriers	ε4-allele Non-carriers	ε4-allele Carriers
	14 (18%)	21 (28%)	21 (28%)	20 (26%)
**Race/Ethnicity %**				
**Black/African American**	0 (0%)	1 (~1.5%)	0 (0%)	1 (~1.5%)
**Caucasian**	6 (8%)	10 (13%)	10 (13%)	10 (13%)
**Hispanic/Latino**	1 (~1.5%)	0 (0%)	0 (0%)	1 (~1.5%)
**Did Not Report**	7 (9%)	10 (13%)	11 (14%)	8 (11%)
**Education %**				
**Some High School**	0 (0%)	1 (~1%)	0 (0%)	1 (~1%)
**High School Degree**	2 (3%)	2 (3%)	3 (4%)	0 (0%)
**Some College**	3 (4%)	6 (8%)	3 (4%)	3 (4%)
**College Degree**	5 (~6%)	2 (3%)	7 (9%)	8 (11%)
**Some Graduate School**	0 (0%)	0 (0%)	0 (0%)	1 (~1%)
**Graduate Degree**	4 (5%)	10 (13%)	8 (11%)	7 (9%)
**General Physical Health Conditions %**				
Allergies/Asthma	2 (3%)	3 (4%)	2 (3%)	3 (4%)
**Cardiovascular**	1 (~1%)	0 (0%)	1 (~1%)	2 (3%)
**Endocrine**	3 (4%)	2 (3%)	1 (~1%)	1 (~1%)
**Gastrointestinal**	0 (0%)	2 (3%)	0 (0%)	0 (0%)
**Cancer**	0 (0%)	1 (~1%)	0 (0%)	0 (0%)
Multiple[a]	6 (8%)	8 (11%)	9 (12%)	4 (5%)
**Did Not Report**	2 (3%)	5 (~7%)	8 (11%)	8 (11%)
**Mental Health Conditions %**				
**Depression**	1 (1%)	3 (4%)	0 (0%)	2 (3%)

**Anxiety**	0 (0%)	2 (3%)	0 (0%)	1 (1%)
**ADHD/ADD**	1 (1%)	1 (1%)	0 (0%)	0 (0%)
**Learning Disorder/Dyslexia**	0 (0%)	0 (0%)	1 (1%)	0 (0%)
**PTSD**	0 (0%)	0 (0%)	1 (1%)	0 (0%)
**Insomnia**	0 (0%)	0 (0%)	1 (1%)	0 (0%)
**Multiple[b]**	8 (11%)	9 (12%)	2 (3%)	2 (3%)
**Did Not Report/NA**	4 (~5%)	6 (8%)	16 (21%)	15 (20%)
**Family History of Alz %**				
**Yes**	1 (1%)	6 (8%)	2 (3%)	1 (1%)
**No**	5 (6%)	8 (11%)	8 (11%)	8 (11%)
***Unknown/ Did Not Report**	8 (11%)	7 (9%)	11 (14%)	11 (14%)

***37 Participants did not report**

[a]Participants had 2–3 comorbid **health diseases**/disorders including **allergies/asthma, gastrointestinal, cancer, bipolar/mood stabilizing, endocrine, cardiovascular, arthritis/joint/bone, and diabetes.**

[b]Participants had 2–3 comorbid **psychiatric disorders** including **ADHD, generalized anxiety disorder, depressed mood, mild cerebral palsy, obsessive compulsive disorder, dysthymia, learning disability, and/or bipolar NOS.**

Supplementary Table S2. AVLT Group Means and SD

	ASD (mean ± SD)			NT(mean ± SD)		
	ε4-allele Non-carriers	ε4-allele Carriers	ε4-allele Combined	ε4-allele Non-carriers	ε4-allele Carriers	ε4-allele Combined
**Short-Term Memory (AVLT[a] A1)**	6.71 (±1.44)	5.67 (±2.29)	6.09 (±2.04)	6.38 (±2.22)	5.60 (±1.57)	6.00 (±1.95)
**Total Words (AVLT[a] A1-A5)**	50.15 (±6.95)	44.86 (±10.95)	46.97 (±9.79)	51.19 (±9.03)	49.05 (±9.12)	50.15 (±9.02)
**Long-Term Memory (A7)**	9.79 (±2.39)	8.67 (±3.35)	9.11 (±3.02)	10.38 (±2.99)	10.30 (±3.25)	10.34 (±3.08)
**Total Words ASD Males**	50.42 (±5.70)	41.87 (±10.74)	45.67 (±9.72)		n/a	

[a]Auditory Verbal Learning Test
